# Metagenomic expansion of Joyebacterota identifies *Cavimicrobium*, a dominant sulfide-producing lineage in anoxic marine ecosystems

**DOI:** 10.1093/ismeco/ycag137

**Published:** 2026-05-17

**Authors:** Xing Chen, Chun-Xu Xue, Jinyan Wang, Shuang Wang, Mengshuai Su, Ronghua Liu, Xiao-Yu Zhu, Jiwen Liu, Peng Yao, Liang Fu, Zuosheng Yang, Chris Greening, Jonathan D Todd, Xiao-Hua Zhang

**Affiliations:** Frontiers Science Center for Deep Ocean Multispheres and Earth System, and College of Marine Life Sciences, Ocean University of China, Qingdao 266003, ShanDong province, China; Frontiers Science Center for Deep Ocean Multispheres and Earth System, and College of Marine Life Sciences, Ocean University of China, Qingdao 266003, ShanDong province, China; Laboratory for Marine Ecology and Environmental Science, Marine Science and Technology Center, Qingdao 266237, ShanDong province, China; School of Food and Drug, Shenzhen Polytechnic University, Shenzhen 518000, GuangDong province, China; Frontiers Science Center for Deep Ocean Multispheres and Earth System, and College of Marine Life Sciences, Ocean University of China, Qingdao 266003, ShanDong province, China; Frontiers Science Center for Deep Ocean Multispheres and Earth System, and College of Marine Life Sciences, Ocean University of China, Qingdao 266003, ShanDong province, China; Frontiers Science Center for Deep Ocean Multispheres and Earth System, and College of Marine Life Sciences, Ocean University of China, Qingdao 266003, ShanDong province, China; Frontiers Science Center for Deep Ocean Multispheres and Earth System, and College of Marine Life Sciences, Ocean University of China, Qingdao 266003, ShanDong province, China; Frontiers Science Center for Deep Ocean Multispheres and Earth System, and College of Marine Life Sciences, Ocean University of China, Qingdao 266003, ShanDong province, China; Frontiers Science Center for Deep Ocean Multispheres and Earth System, and College of Marine Life Sciences, Ocean University of China, Qingdao 266003, ShanDong province, China; Laboratory for Marine Ecology and Environmental Science, Marine Science and Technology Center, Qingdao 266237, ShanDong province, China; Institute of Evolution & Marine Biodiversity, Ocean University of China, Qingdao 266003, ShanDong province, China; Key Laboratory of Marine Chemistry Theory and Technology, Ministry of Education, Ocean University of China, Qingdao 266100, ShanDong province, China; Sansha Track Ocean Coral Reef Conservation Research Institute, Sansha 573199, HaiNan province, China; College of Marine Geosciences, Ocean University of China, Qingdao 266100, ShanDong province, China; Department of Microbiology, Biomedicine Discovery Institute, Clayton, VIC 3800, Australia; Securing Antarctica’s Environmental Future, Monash University, Clayton, VIC 3800, Australia; School of Biological Sciences, University of East Anglia, Norwich Research Park, Norwich, Norfolk NR4 7TJ, United Kingdom; Frontiers Science Center for Deep Ocean Multispheres and Earth System, and College of Marine Life Sciences, Ocean University of China, Qingdao 266003, ShanDong province, China; Laboratory for Marine Ecology and Environmental Science, Marine Science and Technology Center, Qingdao 266237, ShanDong province, China; Institute of Evolution & Marine Biodiversity, Ocean University of China, Qingdao 266003, ShanDong province, China

**Keywords:** novel lineage, blue hole, anoxic environment, sulfite reduction, adaptive evolution

## Abstract

Extreme anoxic environments are hotspots of sulfur cycling and harbor numerous novel uncharacterized microbial lineages. Although the phylum Joyebacterota was recently proposed, its internal phylogenetic architecture and evolutionary adaptations remain poorly understood. Here, we significantly expand the genomic diversity and metabolic framework of this phylum by integrating recovered metagenome-assembled genomes, and propose a novel genus, *Cavimicrobium*. Phylogenomic analysis placed *Cavimicrobium* as a distinct clade and further divided into four species-level subgroups associated with diverse anoxic sources, including sediments from the Salton Sea, the Eastern Gotland Basin, and the anoxic waters of the Sansha Yongle Blue Hole (SYBH). Unlike previous broad surveys, our study revealed that this lineage evolved from a facultatively anaerobic ancestor and underwent adaptive gene gain and loss through phylogenetic reconstruction. Genomic evidence suggested that this lineage harbored a previously overlooked anaerobic sulfite reduction (*asrABC*) pathway that likely mediating thiosulfate uptake and conversion to sulfite and sulfide. Notably, *Cavimicrobium* was particularly abundant in the anoxic waters of SYBH, comprising up to one-third of the bacterial community in particle-associated fraction below 100 m, where it is likely a major contributor to sulfide accumulation. Analysis of MAGs and global amplicon datasets revealed that *Cavimicrobium* is widespread across anoxic environments, comprising up to 0.32% of the bacterial community in 354 200 publicly available 16S rRNA gene amplicon samples. Together, these findings reveal a new lineage dominant in certain anoxic environments where they are likely important mediators of sulfur cycling, and broaden our understanding of biogeochemical potential of Joyebacterota.

## Introduction

Microorganisms represent the most abundant and diverse forms of life on Earth and play a central role in driving global biogeochemical cycles [1]. Uncovering the unique microbial assemblages in extreme environments is essential for resolving the complexities of global biogeochemical cycles and understanding the evolutionary boundaries of life in anoxic habitats. In the past decade, the rapid expansion of sequenced bacterial genomes has facilitated the development of genome-based phylogenies, especially as culture-independent techniques generating thousands of metagenome-assembled genomes (MAGs) from diverse extreme environments, greatly advancing the definition of microbial taxonomy [2], including phylum-level lineages with no cultivated representatives. Nearly complete or complete MAGs recovered from poorly represented or novel microbial lineages have significantly advanced our understanding of the evolutionary processes shaping many bacterial phyla [3–5]. For instance, Gong *et al*. performed phylogenomic analyses on thousands of MAGs derived from 42 marine sediment samples, including those from basins, cold seeps, and hydrothermal vents, and identified 55 MAGs representing four novel bacterial phyla (Blakebacterota, Orphanbacterota, Arandabacterota, and Joyebacterota) as well as one previously proposed phylum (AABM5-125-24), all of which belong to the FCB superphylum [4]. Comparing genomes from diverse habitats and distinct lineages within a given candidate phylum can provide valuable insights into their phylogeny, conserved traits, and metabolic diversity, shedding light on these widespread yet poorly understood branches of the tree of life [5].

One understudied karst bedrock ecosystem enriched with anoxic water is Sansha Yongle Blue Hole (SYBH): a subsurface cave with 300 m depth found in the South China Sea [6]. Typically, the water body in marine blue hole is highly stratified, with anoxic and sulfidic seawater at the deeper depths [7–9], providing a natural marine laboratory to study unusual novel taxa and their potentially important roles in redox-stratified microbial processes. Moreover, substantial gaps remain in our understanding of the active processes within the sulfur cycle in the SYBH, as well as the metabolic pathways and microbial taxa responsible for mediating them. For example, genome-centric metagenomic approaches have greatly expanded the known diversity of potential sulfate and sulfite reducers [10]. Prior studies [11, 12] have revealed distinct vertical profiles of the dominant sulfur-oxidizing bacteria (SOB) and sulfate-reducing bacteria (SRB), along with the functional genes involved in these processes in the SYBH. It was determined that Desulfobacterota, as a typical SRB, exhibited excellent adaptive capacities, utilizing dissimilatory sulfate reduction (via *sat*, *aprAB*, and *dsrAB* genes) to obtain energy in the unique anoxic environment of the deeper SYBH [11]. Despite these significant advances, diversity surveys [13, 14] indicated that key microbial players mediating the marine sulfur cycle remain taxonomically elusive, hindering our ability to accurately assess sulfur flux in stratified anoxic water columns.

In this study, we recovered and reconstructed five high-quality MAGs from anoxic water of the SYBH, and identified them as a previously uncharacterized microbial lineage belonging to the phylum Joyebacterota. Joyebacterota representatives were first discovered and named 3 years ago through reconstruction of 20 MAGs from hydrothermal vent sediments [4]. The definition of this phylum is hindered by the limited number of available uncultivated Joyebacterota genomes, which poses significant challenges for characterizing their metabolic potential and determining their phylogenetic position. Little is currently known about the evolutionary trajectory, ecological distribution, and functional potential of lineages within Joyebacterota. Here, we characterize novel members within the phylum Joyebacterota that exhibit metabolic versatility and broaden ecological distribution, and are likely key mediators of sulfide, particularly in the anoxic waters of the SYBH.

## Materials and methods

### Sampling and environmental factors

Seawater samples were collected from the SYBH (111°768′ E, 16°525′ N) at depths ranging from 0 to 190 m in October 2019. Physicochemical parameters, including salinity, temperature, dissolved oxygen (DO), H_2_S, dissolved organic carbon (DOC), sulfate, and dissolved inorganic nutrients, were measured or analyzed according to previously established protocols [7, 15–18]. For microbial community analysis, one liter of seawater collected from each discrete depth was filtered independently and serially through 3 μm (TSTP, 142 mm, Millipore) and 0.22 μm (GTTP, 142 mm, Millipore) polycarbonate membranes to collect biomass. The 3 and 0.22 μm polycarbonate membranes represent particle-associated (PA) and free-living (FL) fractions, respectively. Microbial biomass was collected from 29 discrete depths for 16S rRNA gene amplicon sequencing to provide a high-resolution profile of the community structure. A subset of these samples, representing seven key hydrographic layers (0, 30, 50, 90, 120, 140, and 170 m), was selected for integrated metagenomic and metatranscriptomic analysis. A larger volume of 50 L per depth was filtered at these seven sites to ensure sufficient biomass and high-quality DNA/RNA recovery for library construction and genomic binning. To ensure transcript stability, these RNA filters were recovered within 30 min of sampling, immediately treated with 1 ml of RNAlater (Ambion, USA), and stored in liquid nitrogen. In contrast, filters for DNA analysis (both amplicon and metagenomic) were directly frozen at −80°C or in liquid nitrogen.

### DNA and RNA extraction and sequencing

Total DNA and RNA for metagenomic sequencing were extracted from the biomass-containing filters representing both fractions at all seven depths, following the protocols described previously [12, 19]. For metagenomics, DNA was extracted using a phenol-chloroform protocol, and Polymerase Chain Reaction (PCR) free libraries were sequenced on the Illumina HiSeq X-Ten platform (2 × 150 bp). For metatranscriptomics, total RNA was extracted (Qiagen RNeasy Mini Kit), reverse-transcribed to cDNA, and sequenced to quantify the active metabolic gene expression of the microbial communities, including the Joyebacterota MAGs recovered in this study. Detailed sequencing and read statistics for both metagenomes and metatranscriptomes are provided in our previous study [12]. Additionally, 16S rRNA gene amplicon sequencing targeting the V4 region (primers 515F/806R) was performed on the Illumina MiSeq PE300 platform across all 29 depths, following the protocols detailed in [12].

### Metagenome assembly, genome reconstruction, and gene annotation

Trimmomatic was used for quality control and trimming of raw reads, including the removal of adapter sequences, low-quality bases, and overly short sequences [20]. After filtering, the clean reads from each sample were individually assembled using MEGAHIT v1.1.2 [21]. Three metagenomic binning software, MaxBin2 v2.2.4 [22], MetaBAT2 v2.12.1 [23], and CONCOCT v0.4.0 [24], were then used to individually bin the assemblies from each sample in the MetaWRAP software [25]. The three final bin sets from the three binning software were then merged into a single, more robust bin set using the minimum completion (-c 50) and maximum contamination (-x 10) parameters that were evaluated by CheckM v1.0.7 [26]. Next, we used the reassemble_bins module within the MetaWRAP software to improve the quality of bins. An approximate taxonomy of MAGs was obtained using Genome Taxonomy Database Toolkit (GTDB-Tk) v0.3.2 [2]. Finally, five *Cavimicrobium* genomes were obtained from 120, 140, and 170 m seawater depths (MAG135-F120 and MAG173-P120 were obtained from 120 m, MAG220-F140 and MAG264-P140 were obtained from 140 m, MAG358-P170 was obtained from 170 m), and 26 *Cavimicrobium* MAGs were obtained from other anoxic environments.

The coding sequences of *Cavimicrobium* genomes were predicted using Prokka v1.14 with default settings and subsequently annotated against the Kyoto Encyclopedia of Genes and Genomes (KEGG) database using the online BlastKOALA tool (KEGG release 100.0, http://www.kegg.jp/blastkoala/) [27]. CAZymes were identified using the online dbCAN2 meta server (http://bcb.unl.edu/dbCAN2), which integrates three complementary tools, HMMER v3.3.2, DIAMOND v2.0.13, and Hotpep, for robust detection of carbohydrate-active enzymes. To further enhance annotation accuracy, we also annotated the coding sequences against the EggNOG v5.0 database using EggNOG-mapper v2.1.9 with default parameters. Genes with ambiguous or uncertain annotations were double-checked using Uniprot (https://www.uniprot.org/) and default NCBI’s Conserved Domain Database (https://www.ncbi.nlm.nih.gov/) (including COG, Pfam, SMART, TIGRFAMs, PRK, and NCBI-curated models). Protein sequences were analyzed using the default search mode (Standard), with an E-value threshold of 1e-5, enabling overlapping domain hits and superfamily classification. Low-complexity regions were masked, and a maximum of 500 hits per query was retained. In addition, AsrABC protein sequences recovered from the metagenomes were compared against the NCBI NR database using BLASTP, and their taxonomic affiliations were analyzed using MEGAN Community Edition v6.20.19 [28].

### Phylogenetic analysis and average acid identity

To infer the phylogenetic position of the recovered MAGs belonging to the Joyebacterota, we employed the GTDB-Tk [29] for standardized taxonomic classification and phylogenomic analysis. The phylogenetic trees were constructed based on the concatenated alignment of 120 bacterial single-copy marker genes (bac120) identified by GTDB-Tk and 16S rRNA genes, respectively. The bac120 were identified and aligned using the identify and align modules of GTDB-Tk [29]. Specifically, marker genes were identified using HMMER, and multiple sequence alignments were generated using FAMSA. To ensure the quality of the phylogenomic inference, the concatenated alignment was filtered using the GTDB-Tk default mask, which removes columns with >50% gaps or low amino acid conservation [29]. 16S rRNA genes were predicted using RNAmmer [30] with default parameters. The 16S rRNA genes of all genomes were aligned using MAFFT v7 [31] with the high-sensitivity (L-INS-i) algorithm and then trimmed using trimAl v1.2 [32] to construct a maximum likelihood (ML) tree using IQ-TREE v1.6.1 [33] with default parameters. The trees were annotated using the Interactive Tree of Life (iTOL) webtool for better visualization [34]. Taxonomic novelty was assessed based on average amino acid identity (AAI) comparisons with publicly available genomes from the GTDB reference tree. Lineages that showed <65% AAI to known genera were considered candidates for novel genus-level taxa [35].

### Calculation of relative abundance

To assess the relative coverage of Joyebacterota MAGs in each metagenomic sample, we used a workflow based on Anvi’o v6.2 [36]. Metagenomic reads were first mapped to the MAGs using Bowtie2 v2.4.2. The resulting alignment files (BAM format) were processed with the anvi-profile program using default settings and a minimum contig length of 1000 bp to compute per-contig coverage statistics. The profiles generated for each sample were then combined using anvi-merge. Finally, the anvi-summarize program was used to extract the average coverage values of each MAG across all metagenomic samples. These average coverage values were used to represent the relative abundance of Joyebacterota MAGs. To estimate the gene-level abundance of functional genes within Joyebacterota MAGs, we used MicrobeCensus v1.1.1 [37] to calculate genome equivalents for each metagenomic sample. The relative abundance of functional genes was quantified using the TPM (transcripts per million) method for both metagenomic (DNA-based) and metatranscriptomic (RNA-based) datasets. TPM was calculated by first normalizing the read counts for gene length (reads per kilobase, RPK) and then scaling by the total RPK within each library [38, 39]. This approach allows for the direct comparison of gene abundance and transcriptional activity across samples with different sequencing depths and gene lengths. Additionally, to determine the relative abundance of genes in the metagenomes, we mapped high-quality reads of each metagenome (generating 14 sam files) to the predicted genes using BWA-MEM (bwa v0.7.15-r1140, MA, USA, using the default setting) [40]. We then used the unsorted sam files as input to pileup.sh (bbmap-38.22-0) to determine the average coverage of each gene.

### Primer design for Quantitative PCR and Reverse TranscriptionQuantitative PCR

The abundance of bacterial 16S rRNA genes was quantified using qPCR, with primers 341F (5′-CCTAYGGGRBGCASCAG-3′) and 802R (3′-TACNVGGGTATCTAATCC-5′). Primers asrA-1 (5′-AAAGAAGCAGAAGGACCTAAG-3′) and asrA-2 (5′-CACTCGCATCATACATATCAGC-3′) used for the anaerobic sulfite reductase subunit A (*asrA*) gene [41] were designed using primer3. The construction of qPCR standards and the qPCR assay was performed as described in a previous study [42]. For reverse-transcription qPCR, 9 μl of purified RNA was mixed with 1 μl random hexamer primers (Invitrogen). The reaction system was incubated at 70°C for 5 min and cooled on ice, before mixing with 1 μl dNTPs (Deoxynucleoside Triphosphate, 10 mM), 4 μl of M-MLV RT 5× buffer (Promega), 0.8 μl of M-MLV reverse transcriptase (Promega), 0.4 μl of RNase inhibitor (40 U μl^−1^, Roche), and 3.8 μl of diethyl pyr-ocarbonate (DEPC) water. This was then incubated at 42°C for 1 h, and the cDNA was stored at −80°C.

### Evaluation of carbon monoxide oxidation activity of *CoxL*

Exponential phase cultures for *Mycobacterium smegmatis* mc2155 wild-type strain and the derived strain Δ*coxL* were maintained on lysogeny broth (LB) agar plates supplemented with 0.05% (w/v) Tween 80. MAG135-F120 *coxL* and WS_11 *coxL* complemented in *M. smegmatis coxL* mutant strains were collected and adjusted to the same OD_600_ = 0.3, then washed twice with 1 ml carbon-free M9 minimal basal medium by centrifugation for 5 min at 4000 rpm and the pellet resuspended in 1 ml carbon-free M9 medium. To a final concentration of 1% (v/v), 120 ml volume flasks containing 20 ml M9 medium (plus 30 μl 50% glycerol as carbon source) were inoculated in triplicate. 1% Carbon Monoxide (CO) (Lsotopes Limited) was added to the headspace of the sealed flasks using a sterile needle and syringe, with the pressure equalized by removing the same volume of air prior to CO addition. The flasks were incubated at 28°C with shaking. The CO concentration in the flask headspace was measured at Day 0 and Day 7 using the PEAK PERFORMER1 manual injection.

### Reconstruction of ancestral genomes

Clusters of homologous proteins were reconstructed for 49 genomes from the Joyebacterota and Eisenbacteria. To ensure data quality, only MAGs meeting the medium or high-quality standards (completeness >50%, contamination <10%) were included in this analysis. The orthologous gene family table was obtained by clustering all of the predicted protein sequences from these genomes using Get_homologues with OrthoMCL (thresholds *E*-value <1e^−10^ and sequence identity ≥30%, and inflation value 1.5) [43]. This yielded a total of 31 153 protein families. The species tree required for ancestral reconstruction was built using IQ-TREE (v1.6.1) based on a concatenated alignment of 120 bacterial marker genes under the LG + F + R10 model. To address the evolution of the metabolic strategies of the Joyebacterota, ancestral family sizes and gene gains and losses were inferred using the program COUNT software with a maximum likelihood birth-and-death model [44, 45]. The rates of gene gain, loss, and duplication were optimized, and the posterior probability of gene presence was calculated at each internal node. The procedure prohibits multiple gains of gene families and allows the reconstruction of gene gain and loss events in both extant strains and potential ancestors.

### Biogeographical distribution

First, the 16S rRNA gene sequences from Joyebacterota genomes were used as queries to search the NCBI nucleotide (nt) database using BLASTn, with a minimum threshold of 90% sequence identity and 50% alignment coverage. The resulting BLASTn hits, along with the original query sequences, were compiled to construct a 16S rRNA reference dataset for subsequent phylogenetic analysis. Secondly, about 354 200 16S rRNA gene amplicon samples from different environments were downloaded from NCBI before August 2021. The reference database was used as a query to be searched with these amplicon samples using the blastn_vdb tool (https://ncbiinsights.ncbi.nlm.nih.gov/2015/12/11/sra-toolkit-the-sra-database-at-your-fingertips/) under strict parameters (similarity ≥99%) to avoid false positives. Following stringent quality filtering and chimera detection via VSEARCH, 1154 samples were retained for the final calculation of relative abundance and global distribution analysis [46]. The operational taxonomic unit (OTU) sequences were then compared to the Joyebacterota 16S rRNA gene reference sequences by BLASTn (97% identity and sequence length ≥ 100 bp). Finally, IQ-TREE v1.6.1 was used for maximum likelihood reconstruction to further determine OTU taxonomy.

## Results and discussion

### Genomic and phylogenetic characterization of a novel lineage within the phylum Joyebacterota

We first selected five uncharacterized genomes [previously classified as a novel phylum VGIX01 in the previous version of the GTDB database (release 220)] from reconstructed 391 MAGs collected from the SYBH [11]. The SYBH exhibited steep physicochemical gradients in dissolved oxygen (from 192 μmol/L at 0 m to levels below the detection limit of the Winkler’s method at 105 m). With the decrease of dissolved oxygen, H_2_S concentration increased from 0.01 μmol/L at 90 m to 251.1 μmol/L at 190 m (Supplementary Table S1). The five high-quality genomes (completeness >96.3%, contamination <1.1%) recovered from 120, 140, and 170 m depths in the anoxic zone of SYBH (Supplementary Table S2). Notably, this lineage was reclassified as the undescribed genus *JAPEKQ01* within the phylum Joyebacterota in the latest GTDB release (release 226). This phylum Joyebacterota was initially proposed and described by Gong *et al*. [4] who first reported its distinct phylogenetic position and metabolic features based on genome-resolved metagenomics. At the time, the phylum comprised only three genera (*Joyebacterium*, *JALGVN01*, and *JALGZG01*), and remained poorly characterized in terms of diversity and ecological function. In this study, we broadly expanded the genomic diversity of Joyebacterota and reconstructed its internal phylogeny, offering a clearer view of the evolutionary structure of this previously underexplored phylum by integrating newly recovered genomes with publicly available MAGs.

Additionally, we reconstructed 23 MAGs from other anoxic marine samples across the world by metagenomic binning that were affiliated with the Joyebacterota, including 14 MAGs from hypersaline lake sediments of the Salton Sea (California), 1 MAG from deep seabed sediments of Gulf of Mexico with hydrocarbon seepage [47], 5 MAGs from sediments of oxygen-deficient water zones in the Baltic Sea Eastern Gotland Basin [48], and 3 MAGs from permanently stratified anoxic seawater of Cariaco Basin [49] (Supplementary Table S2). They had a genome size ranging from 2.35 to 3.14 Mbp and a high GC content (57%–64%). The genome completeness of the MAGs ranged from 86.41% to 98.9%, with low contamination (<10%). Moreover, all additional MAGs belonging to phylum Joyebacterota in the GTDB database were downloaded for subsequent analysis in this study (Supplementary Table S2).

**Figure 1:**
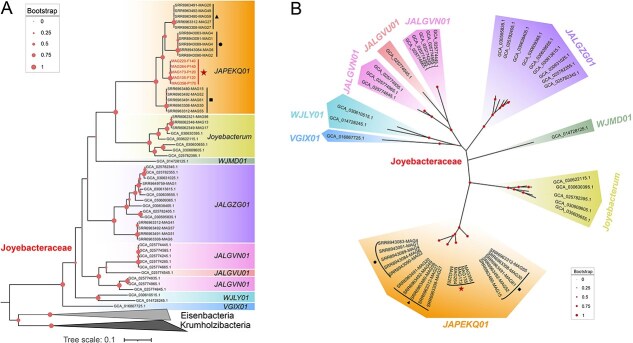
Phylogenetic characterization of a novel lineage within the Joyebacterota. Maximum-likelihood phylogenies of *Cavimicrobium* genomes and related lineages based on the concatenated alignment of 120 bacterial single-copy marker genes (A) and 16S rRNA gene sequences (B). The genome and 16S rRNA gene trees were inferred using IQ-TREE with the best-fit evolutionary models and 1000 bootstrap replicates. Different shapes indicate subgroups within genus *Cavimicrobium* originating from distinct environments. Triangles and squares correspond to two species derived from hypersaline lake sediments in the Salton Sea, California. The pentagram indicates the species identified in this study. Circles represent the species recovered from Eastern Gotland Basin sediments.

Phylogenetic analysis of reconstructed MAGs within the *JAPEKQ01*, based on a concatenated alignment of 120 conserved proteins, placed into a deeply branched, strongly supported bacterial lineage that is distinct from the currently uncultured *Joyebacterum*, *WJMD01*, *JALGZG01*, *JALGVN01*, *JALGVU01*, *WJLY01*, and *VGIX01* lineages, and reference bacterial phyla (Fig. 1A). Interestingly, based on phylogenetic analysis of reconstructed MAGs, a shared root for 10 MAGs from hypersaline lake sediments of the Salton Sea, 5 MAGs from sediments of oxygen-deficient water zones in the Baltic Sea Eastern Gotland Basin, and 5 MAGs from this study suggested that they may constitute the genus *JAPEKQ01* within Joyebacteraceae. Members of the genus *JAPEKQ01* were grouped into four species-level lineages according to their habitat origin. Its precise delimitation requires further 16S rRNA gene phylogeny and AAI analyses. Phylogenetic analysis of the 16S rRNA gene sequences obtained from all MAGs indicated that the *JAPEKQ01* lineage is separated within other unnamed genera (Fig. 1B). AAI is reliable indicator for species circumscription, genomic discreteness, and functional gene content similarity. All MAGs in the phylum Joyebacterota belong to the same family Joyebacteraceae and shared at least 45% AAI similarity. The MAGs were divided into nine distinct lineages at the genus level, with 45%–65% shared AAI similarity. Within genus *JAPEKQ01*, a total of 20 MAGs derived from diverse environments were grouped into four species-level subclusters with 65%–95% shared AAI similarity (Supplementary Table S3). In addition to the previously reported Joyebacterota lineages, identified from hydrothermal vents [4] and cold seeps [50], this study further uncovered diverse members of the phylum from other anoxic environments. These newly recovered MAGs not only expand the known ecological distribution of Joyebacterota but also highlight its broader environmental adaptability and potential roles in biogeochemical cycling across various anoxic habitats.

Overall, the results of the genome, 16S rRNA gene phylogenies, and AAI indicated that these MAGs represented a separate, previously undescribed genus-level lineage for which we proposed to rename as *Cavimicrobium* gen. nov*.* (Ca.vi.mi.cro’bi.um. L. neut. n. *cavum*, a cave or cavity; N.L. neut. n. *microbium*, a microbe; N.L. neut. n. *Cavimicrobium*, a microbe inhabiting a cave or cavity environment), reflecting its origin from a cave, anoxic marine environment. MAG173-P120 [*Cavimicrobium yonglei* (yong’le.i. N.L. gen. n. yonglei, of Yongle, referring to the SYBH)] was selected as the type species of the newly described genus *Cavimicrobium*.

### *Cavimicrobium* adopts an obligately anaerobic heterotrophic lifestyle and possibly contributes to sulfide production

Prior to this study, the metabolic features of Joyebacterota were only briefly characterized based on the detection of novel protein families [4], with evidence suggesting that this phylum were obligate anaerobes encoding extracellular carbohydrate-active enzymes (CAZymes). Our study further confirmed the obligate anaerobic and heterotrophic lifestyle of this lineage. In addition, the presence of diverse functional genes related to biogeochemical cycling revealed a broader range of metabolic potential than previously recognized, suggesting that Joyebacterota may play more versatile and ecologically significant roles within anoxic environments.

**Figure 2:**
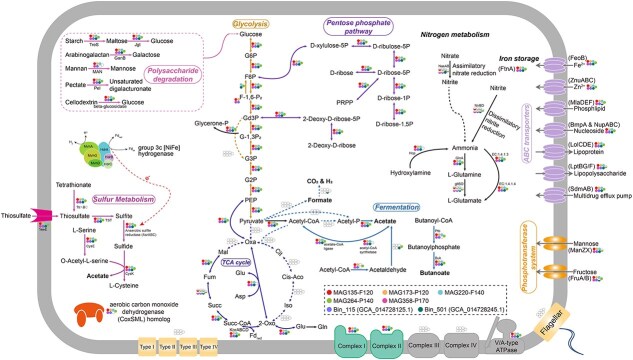
Key metabolic pathways in *Cavimicrobium* MAGs. We reconstructed metabolisms of five MAGs from the Sansha Yongle Blue Hole and two reference genera from hypersaline microbial mats in Shark Bay*.* The presence or absence of enzymes in these pathways is indicated with different dots. Genes associated with the pathways shown in this figure are listed in Supplementary Table S4.

First, we annotated the reconstructed MAGs to predict their metabolic capabilities and biogeochemical roles. The bacteria were predicted to adopt a heterotrophic lifestyle given their genomes lacked any marker genes from the six known carbon fixation pathways (Fig. 2 & Supplementary Table S4). They also lacked any terminal oxidases [i.e. cytochrome c oxidase (*coxABCD*), complex IV (*ccoPNO*) for aerobic respiration, and cytochrome bd oxidase (*cydAB*)], but contained genes for formate metabolism, and acetate fermentation, and thus likely adopt an anaerobic lifestyle (Fig. 2 & Supplementary Table S4). The MAGs potentially encoded complete glycolysis pathways and multiple carbohydrate-active enzymes (CAZymes), including 40 glycosyl hydrolases, potentially enabling them to degrade polysaccharides such as starch, arabinogalactan, mannan, pectate, and cellodextrin (Fig. 2 & Supplementary Table S5). The MAGs from the Eastern Gotland Basin uniquely encoded α-L-fucosidases and chitobiose-degrading enzymes (Supplementary Table S5). Given that fucosidases are primarily derived from marine microbes associated with the degradation of seaweed and that the Eastern Gotland Basin is subject to anthropogenic eutrophication, it is plausible that *Cavimicrobium* populations in this region have adapted to utilize organic carbon derived from sinking algal biomass. Additionally, the MAGs encoded multiple mono/oligosaccharide transporters (Fig. 2). Genes for peptide, oligopeptide, and amino acid transporters were limited in the genomes, suggesting *Cavimicrobium* had limited capacity to use these compounds. All MAGs showed the potential ability to produce acetate directly from acetyl-CoA using acetate-CoA ligase or acetyl-CoA synthetase, and from acetyl phosphate using acylphosphatase or acetate kinase.

*Cavimicrobium* genomes encoded several genes related to sulfur energy metabolism. These bacteria are predicted to import thiosulfate via the YeeE protein, then convert it to sulfite *via* the thiosulfate/3-mercaptopyruvate sulfurtransferase (TST) (Fig. 2 & Supplementary Table S4) [51, 52]. All *Cavimicrobium* MAGs encoded the anaerobic sulfite reductase complex (*asrABC* genes) to reduce sulfite to sulfide (Fig. 2 & Supplementary Table S4). Biochemical studies show the AsrABC catalyzes the production of hydrogen sulfide from sulfite under strictly anoxic conditions [53, 54]. Furthermore, MAGs from Cariaco Basin had the potential to reduce sulfate to sulfite via the sulfate adenylyltransferase (*sat*). These MAGs can either assimilate sulfide into cysteine using cysteine synthase (CysK; cysteine synthase) or release the compound directly. Reductant derived from hydrogen (H_2_) oxidation likely supports sulfate reduction. Consistently, all MAGs encoded the group 3c [NiFe]-hydrogenase (*mvh* complex) known to couple H_2_ oxidation to heterodisulfide reduction in methanogens [55, 56]. Given the lack of methanogenic pathways in *Cavimicrobium* MAGs, we propose that the released electrons could be transferred to HdrABC to reduce heterodisulfides and then to the sulfite reductase complex for sulfite reduction (Supplementary Table S4). In addition, the MAGs contained gene clusters encoding enzymes from the molybdenum-containing hydroxylase superfamily distantly related to the aerobic carbon monoxide dehydrogenase (*coxLMS*). However, our extensive complementation experiments suggested that these enzymes do not consume carbon monoxide as their primary substrate and likely function in a distinct as-yet unresolved role (Supplementary Note 1). We also observed genes for adaptation to their saline to hypersaline niches, including sodium-translocating V-type ATPases and Nqr-type NADH dehydrogenases.

### *Cavimicrobium* is an abundant sulfide producer in the SYBH anoxic waters

**Figure 3:**
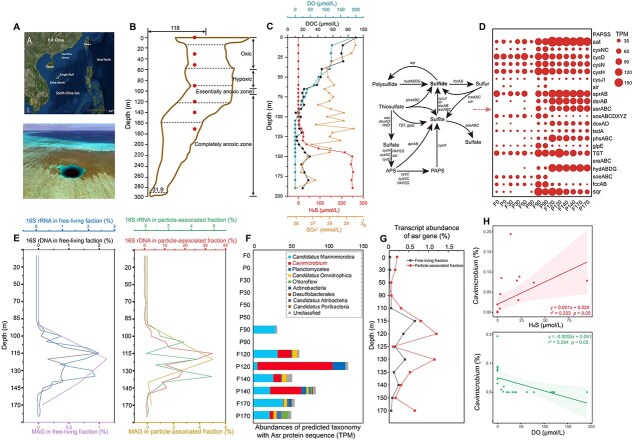
*Cavimicrobium* is a probable abundant sulfide producer in the SYBH anoxic waters. (A) Satellite image of the South China Sea from the Environmental Systems Research Institute (ESRI) and aerial image of the SYBH from an unmanned aerial vehicle. (B) Vertical geometry of the SYBH. (C) Concentrations of total dissolved oxygen, dissolved organic carbon, H_2_S, and SO_4_^2−^ along the depth of the SYBH. (D) Relative abundance of *Cavimicrobium* based on MAGs, 16S rRNA gene, and 16S rRNA sequencing in free-living and particle-associated factions. The mean of a *Cavimicrobium* MAG was estimated by summing the coverage of each nucleotide and dividing by the length of the MAG, then normalizing to the total number of genomes in each sample. (E) Relative abundance of genes involved in the microbial sulfur cycle along the depth in the SYBH. (F) Predicted taxonomy of Asr protein derived from the metagenomes. (G) Transcript abundance of the *Asr* gene was estimated by RT-qPCR over the depth. (H) Correlations between the relative abundance of *Cavimicrobium* and DO and H_2_S concentration from 0 to 140 m.

To better understand the ecological importance of *Cavimicrobium* in sulfur cycling, we investigated their distribution along the depth profile of the SYBH through metagenomic read mapping and 16S rRNA gene amplicon sequencing (Fig. 3A–D). *Cavimicrobium* was mostly absent (<0.01% in most depths) in the aerobic zone (0–90 m) and only present in the essentially anoxic zone and completely anoxic zone (below 90 m) (Fig. 3E & Supplementary Table S6). 16S rDNA gene amplicon sequencing showed that relative abundance of *Cavimicrobium* in particle-associated fraction reached an astonishing ~33.5% at 115 m depth. The relative abundance of *Cavimicrobium* MAGs in the metagenomic data reached ~5.0% at 120 m depth. In addition, the relative abundance of 16S rRNA from cDNA template amplicon sequencing reached ~6.0% at 115 m and 140 m in particle-associated fraction, confirmed that *Cavimicrobium* was highly transcriptionally active in these waters.

To assess whether *Cavimicrobium* was an important sulfite reducer in the SYBH, we analyzed the abundance and affiliations of sulfur cycling genes present in the metagenomic data (Fig. 3D). Sulfur reduction was predicted to be the dominant process in deeper waters, with gene encoding sulfite reductase (*asrABC* and *dsrAB*), sulfate adenylyltransferase (*sat*), adenosine 5′-phosphosulfate reductase (*aprAB*), and thiosulfate reductase (*phsABC*) exhibiting higher abundance in the anoxic zones. The relative abundance of functional genes in sulfur metabolic processes was variably correlated with environmental factors: sulfate reduction exhibited inverse correlation with environmental factors compared to sulfur oxidation and was significantly correlated with temperature, salinity, dissolved oxygen, H_2_S, NH_4_^+^, and dissolved organic carbon (Fig. S1).

Based on our previously reported analysis [11], sulfur oxidation processes involving S^0^, HS^−^, and H_2_S were likely mediated mainly by Rhodobacteraceae, Ectothiorhodospiraceae, and Chromatiaceae in oxic waters and lineages such as Chlorobia in anoxic waters. In contrast, Desulfobacterota encoding dissimilatory sulfite reductase (*dsrABC*) were main sulfate-reducing bacteria below 140 m. The *dsrABC* genes exhibit high efficiency in the reduction process within the respiratory chain, where sulfate serves as the electron acceptor. However, genomic analysis revealed that all 20 *Cavimicrobium* MAGs lack the *dsrAB* genes but harbor the *asrABC* gene cluster (encoding anaerobic sulfite reductase), which is responsible for the reduction of sulfite to sulfide. This suggests a divergent strategy for sulfur transformation compared to other FCB members. While the absence of *dsrAB* suggests that *Cavimicrobium* does not perform canonical sulfate reduction, the presence of a complete *asrABC* identifies it as a specialized sulfite reducer. In complex anoxic systems, sulfite-reducing microorganisms act as essential linkers in the sulfur cycle, utilizing intermediates that are often transient but energy-rich. This metabolic specialization may explain their dominance in the anoxic waters of the Blue Hole, where they could bypass the multi-step sulfate reduction process and directly contribute to the sulfide pool. Indeed, the *asrABC* sequences at 120 and 140 m depth mainly affiliated with the *Cavimicrobium* (Fig. 3F). The high transcriptional activity of the *asrABC* gene was also abundantc in 120 and 140 m (Fig. 3G), consistent with the distribution of the *Cavimicrobium*. In addition, the abundance of *Cavimicrobium* was significant negatively correlated with dissolved oxygen (*r*^2^ = 0.264, *P* < .05) and significant positively correlated with H_2_S (*r*^2^ = 0.233, *P* < .05) above 140 m (Fig. 3H). In the anoxic zones, *Cavimicrobium* may utilize sulfite derived either from the abiotic reaction between upward-diffusing H_2_S and oxidants at the redox interface or from sulfite produced by nearby sulfate-reducing bacteria. Altogether, these results indicated that the *Cavimicrobium* may potentially play a significant ecological role in the sulfur cycling, especially in the anaerobic conversion of sulfite to sulfide in the YBH completely upper anoxic zone.

### *Cavimicrobium* evolved from a facultatively anaerobic ancestor and was diverged through adaptive gene gain and loss

**Figure 4:**
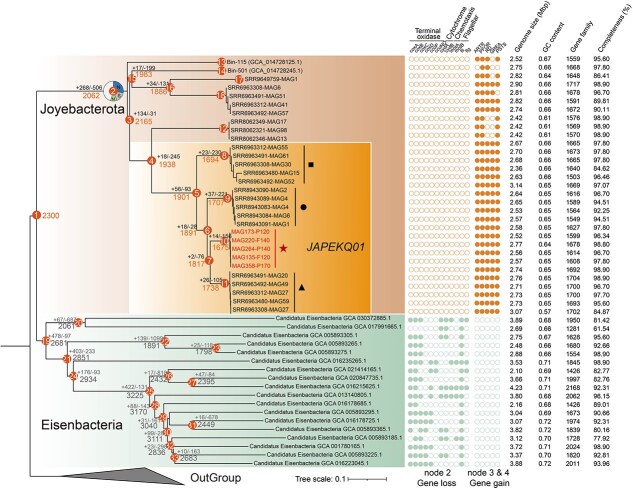
Reconstruction of ancestral genome of *Cavimicrobium*. This is based on a maximum likelihood phylogeny of the *Cavimicrobium*, Eisenbacteria, and outgroup lineages (collapsed). The number next to each node is the number of gene families, gene gained, and gene lost predicted for that node; the pie chart represents the approximate ratio of COG functional annotation classifications that were lost at node 2. The genomic features such as genome size, GC content, the number of gene families, predicted genome completeness are shown next to each taxon. S: function unknown; N: cell motility; C: energy production and conversion; M: membrane/envelope biogenesis; E: amino acid transport and metabolism; G: carbohydrate transport and metabolism; ARTR, anaerobic ribonucleoside-triphosphate reductase; PFOR, pyruvate:ferredoxin oxidoreductase; RD, reductive dehalogenase; GREA, glycyl-radical enzyme activating protein; PSTS, phosphorelay signal transduction system.

To explore how *Cavimicrobium* evolved from its common ancestor with Eisenbacteria, we reconstructed phylogenomic relationships and inferred gene gain and loss events using the birth-and-death model in the COUNT software (Fig. 4 & Supplementary Table S7). Ancestral genome reconstruction inferred that the last common ancestor (LCA) of the *Cavimicrobium* and Eisenbacteria carried a genome with 2300 gene families. The nonrandom gene gains and losses were indicative of adaptive evolution and niche differentiation for *Cavimicrobium* populations (Supplementary Note 2). This genome encodes terminal oxidases for aerobic respiration, fermentation pathways (e.g. for acetate and formate production), carbohydrate-active enzymes (e.g. for chitobiose and cellodextrin degradation), and the aforementioned genes for thiosulfate conversion to sulfide (e.g. *yeeE*, *tst*, *asrABC*). These findings suggest that the LCA of *Cavimicrobium* and Eisenbacteria adopted a chemoorganotrophic and facultatively anaerobic lifestyle. This ancestor evolved into the LCA of *Cavimicrobium* and Eisenbacteria through distinct processes.

The transition to the *Cavimicrobium* LCA was associated with the loss of 506 gene families at node 2 (Fig. 4 & Supplementary Table S7). This includes the loss of the terminal oxidases, resulting in the transition of *Cavimicrobium* from a facultative to obligate anaerobe. Concurrently, these microbes lost their capacity for motility and chemotaxis. Cell motility is widely suggested as an important trait for copiotrophs in aquatic habitats, and this gene loss likely suggests *Cavimicrobium* transitioned to a niche-restricted oligotrophic lifestyle. As elaborated in Supplementary Note 3, the phylogenomic tree nevertheless suggests the phylum also acquired genes during its subsequent proliferation in different anoxic niches, for example, pyruvate:ferredoxin oxidoreductase involved in fermentation, Nqr for sodium-dependent energy conservation, and various environmental sensing systems.

### *Cavimicrobium* is globally distributed in marine anoxic environments

**Figure 5:**
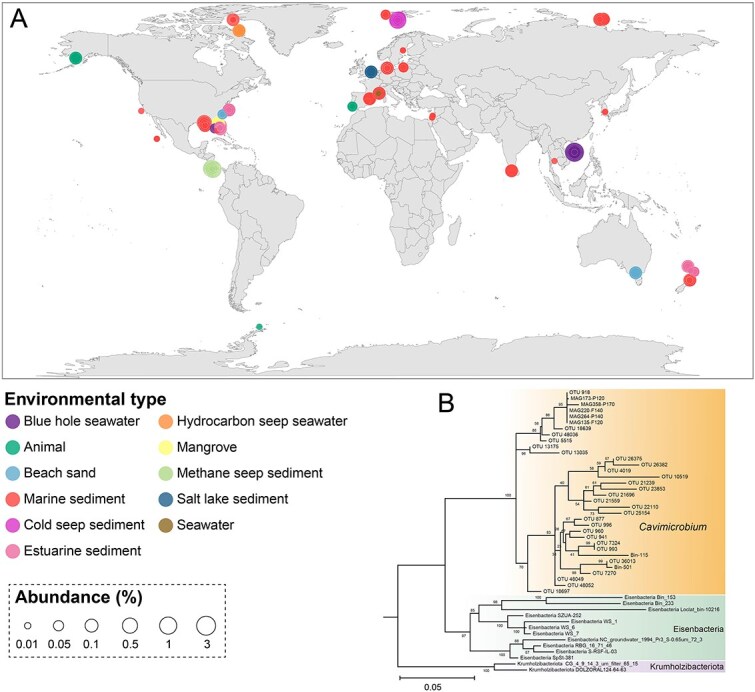
*Cavimicrobium* is globally distributed in marine anoxic environments. (A) Distribution of *Cavimicrobium* 16S rRNA genes in various environments worldwide, shown in different colors. Detailed information of the 16S rRNA genes and samples containing *Cavimicrobium* can be found in Supplementary Tables S8 and S9. (B) The phylogenetic tree of a total of 27 *Cavimicrobium* OTUs alongside Eisenbacteria and Krumholzibacteriota.

Analysis of previously published metagenomes and amplicon sequences suggests *Cavimicrobium* is widely distributed. We recovered other 15 *Cavimicrobium* MAGs mainly from sediments through analysis of 673 global metagenomic samples (Supplementary Table S8). In addition, to further assess the global distribution of *Cavimicrobium*, we examined ~354 200 published 16S rRNA gene amplicon samples from various environments using blast_vdb with a strict cutoff of ≥99%. Although this threshold is likely underestimated their abundance, *Cavimicrobium* was detected in 1154 samples, representing ~0.33% of the ~354 200 surveyed samples examined (Fig. 5A & Supplementary Table S9). A total of 27 *Cavimicrobium* OTUs were found (Fig. 5B & Fig. S2), according to the species-level delineation sequence similarity threshold of 98.65% [57]. The environments containing *Cavimicrobium* included blue hole anoxic seawater, anoxic estuarine sediment, hydrothermal vents with high levels of H_2_S, cold seep sediments and waters rich in hydrocarbons, salt marsh sediments, and mangroves sediments, indicating this phylum is well adapted to anoxic marine environments (Fig. 5A & Supplementary Table S9). Additionally, *Cavimicrobium* 16S rRNA gene sequences were also found in glacial soils and various parts of marine animals, such as cetacean (*Delphinapterus leucas*) skin, sea urchin (*Abatus agassizii*) gut, and clam digestive glands (Supplementary Table S9). The relative abundance of *Cavimicrobium* was highest in the SYBH (>3%), followed by methane seep sediments (1%–2%). The physicochemical properties of the environments containing *Cavimicrobium* varied considerably, but most were anaerobic. These data show that *Cavimicrobium* is widely distributed worldwide and is likely to play important ecological roles in anaerobic environments.

## Conclusion

In this study, our findings significantly extended the metabolic framework of Joyebacterota beyond the initial descriptions provided by a previous study. While earlier research identified the presence of this phylum in anoxic environments, our high-resolution analysis of *Cavimicrobium* revealed a distinct ecological strategy centered on anaerobic sulfite reduction. Crucially, we identified the *asrABC* genes as a core functional feature, a detail that was overlooked in earlier broad-scale genomic surveys. The evolutionary analysis via gene gain and loss further distinguished our work that *Cavimicrobium* evolved from a facultatively anaerobic ancestor and underwent adaptive gene gain and loss, which likely contributed to its key ecological role in local sulfide production in anoxic SYBH. By linking these genomic features to their global distribution, we provide a mechanistic explanation for why this lineage is particularly successful in the anoxic sulfide-rich layers of the SYBH compared to other marine habitats. Together, these findings broaden the genomic diversity and ecological function of Joyebacterota, and provide novel insights into the evolutionary adaptation of microbial lineages to extreme anoxic environments.

## Supplementary Material

Supplemental_metarial_ycag137

Supplementary_tables_ycag137

## Data Availability

All sequence data are archived in the NCBI database under BioProject PRJNA822069. Genome bins can be found at NCBI under the Accession numbers SAMN27169314 (MAG135-F120), SAMN27169315 (MAG173-P120), SAMN27169316 (MAG220-F140), SAMN27169317 (MAG264-P140), and SAMN27169318 (MAG358-P170). Raw reads were deposited in the national omics data encyclopedia (NODE) under BioProject number OEP003486.
