# Supplementary material for: Metagenomic expansion of Joyebacterota identifies Cavimicrobium, a dominant sulfide-producing lineage in anoxic marine ecosystems
Source: ISME Commun. 2026 May 17;6(1):ycag137. doi: 10.1093/ismeco/ycag137 (PMC13271369; doi:10.1093/ismeco/ycag137)
Supplement: Supplemental_metarial_ycag137 [file supplemental_metarial_ycag137.docx]

Supplementary materials for

Metagenomic expansion of Joyebacterota identifies *Cavimicrobium*, a dominant sulfide-producing lineage in anoxic marine ecosystems

**Supplementary note 1**

***Cavimicrobium* encodes molybdenum-containing hydroxylases that lack carbon monoxide oxidation function**

*Cavimicrobium* and the related phylum Eisenbacteria WS_11 encoded complete candidate gene clusters of putative molybdenum-containing hydroxylases homologous to the aerobic-type carbon monoxide dehydrogenase (CODH) enzyme (*coxLMS*) (Fig. S2 $ S3A) known to support CO-dependent autotrophic growth and mixotrophic persistence^[^[^1^](#_ENREF_1)^,^ [^2^](#_ENREF_2)^,^ [^3^](#_ENREF_3)^,^ [^4^](#_ENREF_4)^,^ [^5^](#_ENREF_5)^]^. However, the *Cavimicrobium* and Eisenbacteria putative CoxL (CODH large subunit), CoxS (CODH small subunit) and CoxM (CODH medium subunit) proteins shared only 30.2-34.7%, 43.2-57.5% and 35.8-36.8% amino acid identity, respectively, with their structurally characterized equivalents from the model carboxydotroph *Oligotropha carboxidovorans* (Fig. S3A). The predicted structures of the *Cavimicrobium* putative CoxLMS were highly similar to their equivalents in *O. carboxidovorans* with root-mean-square deviation (RMSD) values between 0.809 to 2.317 depending on the subunit (Fig. S3CDE). These predicted structures included the conserved active and metal-binding (2Fe-2S) sites for CoxS, the active and FAD-binding sites for CoxM, and molybdenum-binding site for CoxL, that are key for CO oxidation in *O. carboxidovorans* (GO:0030151)^[^[^6^](#_ENREF_6)^]^. As *coxLMS* had previously only been found in aerobic or facultatively anaerobic bacteria and archaea^[^[^5^](#_ENREF_5)^]^, it was important to assess the activity of this putative CODH enzyme in these anaerobic bacteria, specifically CoxL. To evaluate the CODH activity of these putative CoxL proteins from *Cavimicrobium* and Eisenbacteria, the respective *coxL* genes were synthesized, cloned into the PLMB509 vector, and used to complement the CODH activity lost in a previously described *M. smegmatis* mc^2^155 *coxL* mutant strain (Fig. S3B). Cloned *Cavimicrobium* and Eisenbacteria *coxL* to the *M. smegmatis* mutant strain conferred no or extremely low CODH activity (Fig S3B). Altogether, this suggests that *Cavimicrobium* encodes novel molybdenum-containing hydroxylases that use an as-yet-unidentified substrate rather than CO.

**Method**

### Protein *in silico* modeling and analysis of natural selection

Structural models of the potential CODH subunits CoxS, CoxM and CoxL from MAG135-F120 were predicted using the ColabFold by combining the homology search of MMseqs2 with AlphaFold2 on default settings^[^[^7^](#_ENREF_7)^]^. Individual CoxLMS subunits were aligned to the confirmed structure of *O. carboxidovorans* OM5 CODH using PyMOL v2.5.2^[^[^8^](#_ENREF_8)^]^.

**References**

1. Hedlund BP, Dodsworth JA, Murugapiran SK, Rinke C, Woyke T. Impact of single-cell genomics and metagenomics on the emerging view of extremophile “microbial dark matter”. *Extremophiles* **18**, 865-875 (2014).

2. Cunliffe M. Correlating carbon monoxide oxidation with *cox* genes in the abundant marine Roseobacter clade. *The ISME journal* **5**, 685-691 (2011).

3. Dobbek H, Gremer L, Kiefersauer R, Huber R, Meyer O. Catalysis at a dinuclear [CuSMo(O)OH] cluster in a CO dehydrogenase resolved at 1.1-Å resolution. *Proceedings of the National Academy of Sciences* **99**, 15971-15976 (2002).

4. King GM, Weber CF. Distribution, diversity and ecology of aerobic CO-oxidizing bacteria. *Nature Reviews Microbiology* **5**, 107-118 (2007).

5. Cordero PR*, et al.* Atmospheric carbon monoxide oxidation is a widespread mechanism supporting microbial survival. *The ISME journal* **13**, 2868-2881 (2019).

6. Schübel U, Kraut M, Mörsdorf G, Meyer O. Molecular characterization of the gene cluster coxMSL encoding the molybdenum-containing carbon monoxide dehydrogenase of Oligotropha carboxidovorans. *Journal of bacteriology* **177**, 2197-2203 (1995).

7. Mirdita M, Schütze K, Moriwaki Y, Heo L, Ovchinnikov S, Steinegger M. ColabFold-Making protein folding accessible to all. *bioRxiv*, (2021).

8. DeLano WL. Pymol: An open-source molecular graphics tool. *CCP4 Newsl Protein Crystallogr* **40**, 82-92 (2002).

**Supplementary note 2**

**Genomic adaptive evolution in *Cavimicrobium*: gain and loss patterns**

We examined reconstructed MAGs to create a genomic profile of *Cavimicrobium*. The phylogenomic trees of *Cavimicrobium* indicated that MAGs from the same location tended to cluster together within clades based on genomic content similarity, and MAGs from sediments clustered separately into different clades. At the tip of the phylogeny (node 2), Joyebacterota lineages initially undergo genomic reduction, subsequently followed by genomic expansion. A basal Joyebacterota lineage, represented by genome Bin-115, escaped the early surge and evolved directly into a highly reduced genome with only 1,559 genes. In contrast, the remaining *Cavimicrobium* clades experienced a gradual trend towards genome reduction following an initial period of rapid innovation (Fig. 4). These suggested three significant evolutionary stages in Joyebacterota: an early period marked by substantial gene losses and gains along the branch node 2; the second occurring, marked by abrupt genomic reduction (Bin-115); and a more recent period is defined by ongoing gene loss along the branches, culminating in the *Cavimicrobium* groups. These nonrandom collection of Joyebacterota gene functions gained was indicative of adaptive evolution, and later genomic and metabolic streamlining were the primary evolutionary processes influencing the divergence of *Cavimicrobium*. Functional annotation of gene families based on COGs indicated that putative biological functions gained and lost during the evolution of the *Cavimicrobium* were significantly different (Fig. 4). Along the LCA node 2, lost genes were primarily biased toward cell motility and cell membrane biogenesis. And the genes predominantly acquired were associated with unknow functions, energy production and conversion. Four COGs categories annotation also revealed functional differences of gained and lost genes at nodes of *Cavimicrobium* subgroups (Fig. S4). The subgroups exhibit a sequential decrease in the number of lost genes across the four COGs categories. Therefore, these non-random gene gains and losses were indicative of adaptive evolution and niche differentiation for *Cavimicrobium* populations.

**Supplementary note 3**

**Efficient energy utilization of oligotrophic *Cavimicrobium* for survival under anaerobic condition**

The genes loss and non-motility at node 2 facilitated the *Cavimicrobium* to oligotrophs (Fig. 4 & Supplementary Table S7). One common way of categorizing heterotrophic marine bacteria life strategies is copiotrophic or oligotrophic^[1]^. Cell motility is widely suggested as an important trait for copiotrophs in aquatic habitats^[^[^2^](#_ENREF_36)^]^. Oligotrophs have proportionately fewer transcriptional regulatory genes than copiotrophs and are generally non-motile/chemotactic^[^[^2^](#_ENREF_36)^]^. A significant number of bacterial flagellin genes (*flh*/*fli*/*flg*) associated with cell motility and chemotaxis genes (*mot*/*che*) were lost in the early evolution of *Cavimicrobium*. *Cavimicrobium* also lacked or simplified certain genes, such as high-affinity substrate transport systems (including specific transport proteins for amino acids, sugars, ions, and more) (Supplementary Table S7), which were typically used for rapid uptake in nutrient-rich environments with high substrate concentrations. This loss or simplification of genes was an evolutionary adaptation that enabled oligotrophs to efficiently utilize limited nutrients in resource-scarce environments. Additionally, the absence of the motility trait in oligotroph *Cavimicrobium* may be linked to small genomic size that is proposed to be part of a suite of adaptations^[^[^1^](#_ENREF_35)^]^. These results indicated that the *Cavimicrobium* ware oligotrophs with non-chemotactic, and did not experience adaptive evolution to environmental changes, likely due to geographic isolations.

Gene gain at node 3 facilitated alternative electron acceptors expansion for efficient energy (Fig. 4 & Supplementary table S7). Numerous crucial genes involved in ATP synthesis, electron transport chains, and cellular energy metabolism were acquired at nodes 3. For example, anaerobic ribonucleoside-triphosphate reductase utilized specific electron donors (H_2_S and CH_4_) to maintain adequate levels of dNTPs in microbes under anaerobic conditions^[^[^3^](#_ENREF_38)^]^, thereby supporting *Cavimicrobium* growth and survival in oxygen-deprived environments. Gained pyruvate: ferredoxin oxidoreductase (PFOR) is a key and highly oxygen-sensitive enzyme in strictly bacterial anaerobic metabolism, such as catalyzing the oxidative decarboxylation of pyruvate to acetyl-CoA and CO_2_^[^[^4^](#_ENREF_39)^]^. PFOR also contained one to three 4Fe-4S clusters that were especially widespread in anaerobes and facilitated electron transfer^[^[^5^](#_ENREF_39)^]^. Acetate kinase family typically converted acetate to acetyl phosphate under anaerobic or oxygen-limited conditions^[^[^5^](#_ENREF_40)^]^, thereby contributing to *Cavimicrobium* energy generation processes. Gained formate acetyltransferase glycine radical converts formate to acetate under conditions of elevated formate concentration or oxygen limitation. Reductive dehalogenase utilized organic halogen compounds (such as chlorinated and brominated alkanes) as reducible electron acceptors to generate energy in anaerobic or low-oxygen environments^[^[^6^](#_ENREF_41)^]^. Additionally, the NQR complex (*nqrA* and *nqrC*) relied on other redox reactions under anaerobic conditions to facilitate the translocation of sodium ions (Na^+^) across the cell membrane, supporting *Cavimicrobium* energy metabolism and electron transfer^[^[^7^](#_ENREF_42)^]^.

Gene gain enhanced environmental sensing and response capabilities in *Cavimicrobium* (Fig. 4 & Supplementary table S7). For example, the phosphorelay signal transduction system sensed environmental signals such as temperature, nutrient concentrations, oxygen levels, and others, enabling responses to changes in the external environment^[^[^8^](#_ENREF_43)^]^. The glycyl-radical enzyme activating protein family^[^[^9^](#_ENREF_44)^]^ was predominantly involved in activating a specialized group of energy metabolism enzymes and environmental stress response enzymes, particularly under anaerobic conditions. Gained histidine kinase was a membrane protein that senses environmental signals^[^[^10^](#_ENREF_45)^]^. Upon detecting changes in oxygen concentration, it adapted to anaerobic conditions by potentially regulating gene expression associated with redox balance.

**References**

1. Noell, S.E., et al., *Differences in the regulatory strategies of marine oligotrophs and copiotrophs reflect differences in motility.* Environmental Microbiology, 2023. **25**(7): p. 1265-1280.

2. Lauro, F.M., et al., *The genomic basis of trophic strategy in marine bacteria.* Proceedings of the National Academy of Sciences, 2009. **106**(37): p. 15527-15533.

3. Fontecave, M., R. Eliasson, and P. Reichard, *Oxygen-sensitive ribonucleoside triphosphate reductase is present in anaerobic Escherichia coli.* Proceedings of the National Academy of Sciences, 1989. **86**(7): p. 2147-2151.

4. Chabriere, E., et al., *Crystal structure of the free radical intermediate of pyruvate: ferredoxin oxidoreductase.* Science, 2001. **294**(5551): p. 2559-2563.

5. Pan, X., et al., *Deep insights into the network of acetate metabolism in anaerobic digestion: focusing on syntrophic acetate oxidation and homoacetogenesis.* Water Research, 2021. **190**: p. 116774.

6. Payne, K.A.P., et al., *Reductive dehalogenase structure suggests a mechanism for B12-dependent dehalogenation.* Nature, 2015. **517**(7535): p. 513-516.

7. Ito, T., et al., *Genetic and Biochemical Analysis of Anaerobic Respiration in Bacteroides fragilis and Its Importance In Vivo.* mBio, 2020. **11**(1): p. 10.1128/mbio.03238-19.

8. Hoch, J.A. and K.I. Varughese, *Keeping Signals Straight in Phosphorelay Signal Transduction.* Journal of Bacteriology, 2001. **183**(17): p. 4941-4949.

9. Sawers, G., *Biochemistry, physiology and molecular biology of glycyl radical enzymes.* FEMS microbiology reviews, 1998. **22**(5): p. 543-551.

10. Kim, D.-j. and S. Forst, *Genomic analysis of the histidine kinase family in bacteria and archaea.* Microbiology, 2001. **147**(5): p. 1197-1212.

**Supplementary Figures**


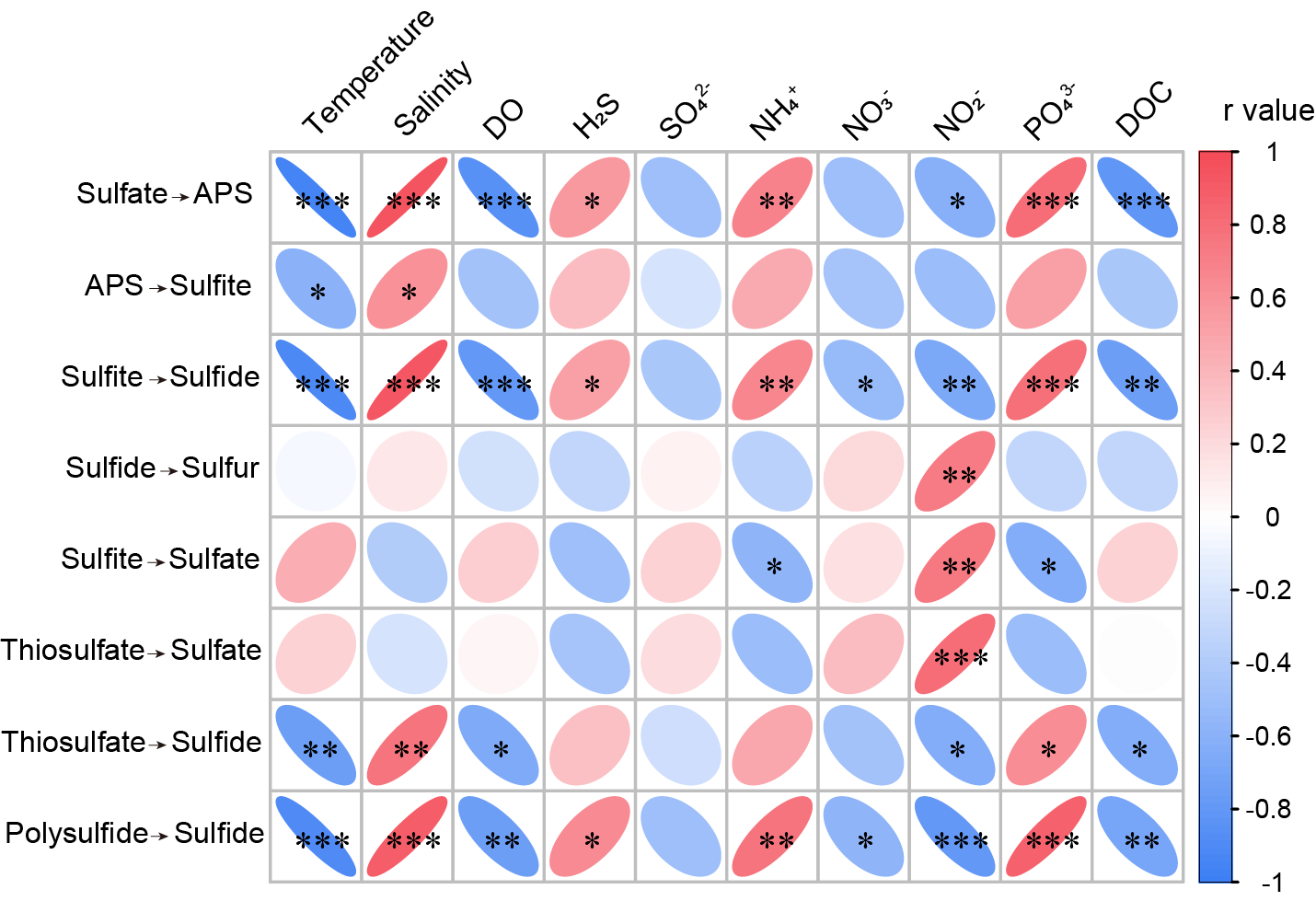


**Fig. S1** The correlation between main environmental factors and sulfur oxidation, sulfate reduction in the Sansha Yongle Blue Hole.


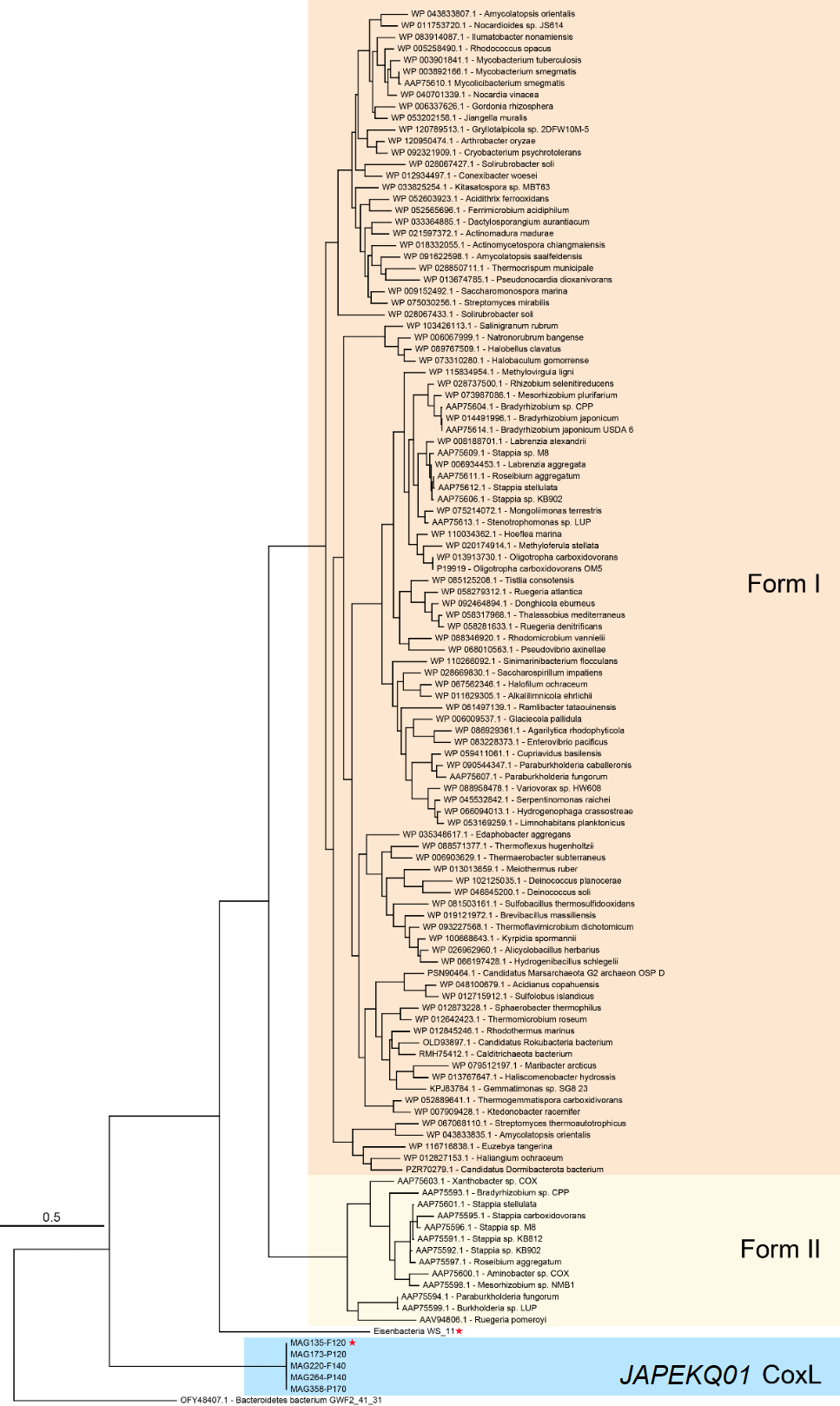


**Fig. S2** The phylogenetic tree of CoxL proteins with more form I sequences. The function of CoxL sequences marked with red star in the tree were verified in this study. The reference sequences of CoxL proteins were achieved from the study^1^.

**Reference**

1. Cordero PR, Bayly K, Man Leung P, Huang C, Islam ZF, Schittenhelm RB, King, *et al* (2019). Atmospheric carbon monoxide oxidation is a widespread mechanism supporting microbial survival. *The ISME journal* 13: 2868-2881.


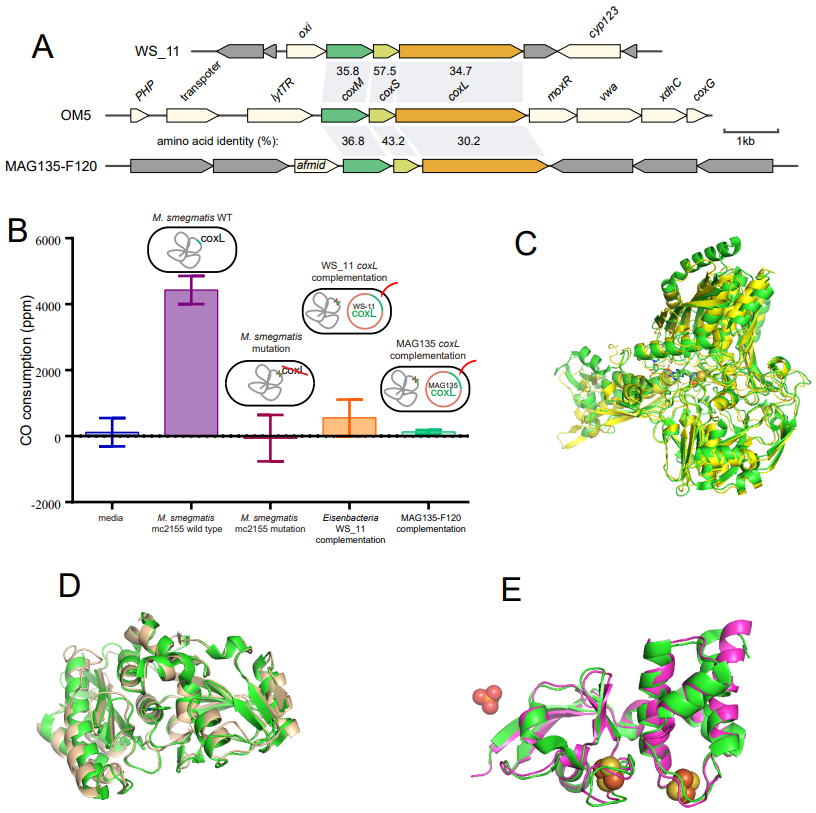


**Fig. S3** **(A)** Genetic neighbourhood of the *coxLMS* gene cluster in Eisenbacteria WS_11, *O. carboxidovorans* OM5 and *Cavimicrobium* MAG135-F120. Abbreviations of gene names are shown in italics. *oxi*, putative oxidoreductase; *cyp123*, putative cytochrome P450 123; *PHP*, PHP domain-containing protein; *lytTR*, LytTR family transcriptional regulator; *moxR*, MoxR family ATPase; *vwa*, VWA domain-containing protein; *xdhC*, XdhC family protein; *coxG*, protein that forms a transient association with CoxLMS and carries menaquinone; *afmid*, kynurenine formamidase. **(B)** Carbon monoxide consumption by wild type *M. smegmatis* mc^2^155, *M. smegmatis* mc^2^155 with *coxL* mutation, *Cavimicrobium* MAG135-F120 and Eisenbacteria WS_11 *coxL* complemented in *M. smegmatis* mc^2^155 *coxL* mutant strains. Superposition of the MAG135-F120 predicted structure model on the ligand binding sites of the OM5 CoxL **(C)**, CoxM **(D)**, and CoxS **(E)** subunits.


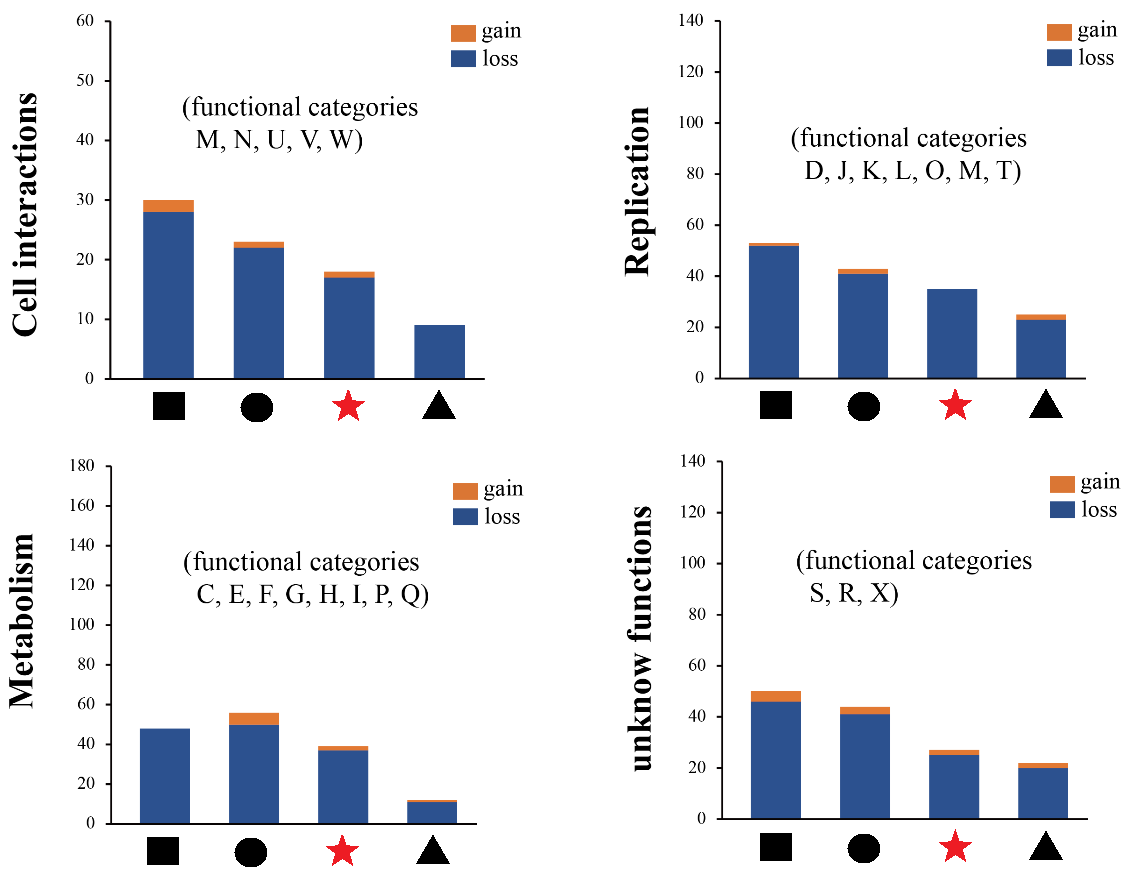


**Fig. S4** Four COGs categories annotation represented functional differences of gained and lost genes at nodes of *Cavimicrobium* subgroups.
